# Dose-response characteristics of methylphenidate on locomotor behavior and on sensory evoked potentials recorded from the VTA, NAc, and PFC in freely behaving rats

**DOI:** 10.1186/1744-9081-2-3

**Published:** 2006-01-17

**Authors:** Pamela B Yang, Alan C Swann, Nachum Dafny

**Affiliations:** 1 Department of Neurobiology and Anatomy, The University of Texas-Medical School at Houston, P.O. Box 20708, Houston, Texas 77225, USA; 2 Department of Psychiatry and Behavioral Sciences, The University of Texas-Medical School at Houston, P.O. Box 20708, Houston, Texas 77225, USA

## Abstract

**Background:**

Methylphenidate (MPD) is a psychostimulant commonly prescribed for attention deficit/hyperactivity disorder. The mode of action of the brain circuitry responsible for initiating the animals' behavior in response to psychostimulants is not well understood. There is some evidence that psychostimulants activate the ventral tegmental area (VTA), nucleus accumbens (NAc), and prefrontal cortex (PFC).

**Methods:**

The present study was designed to investigate the acute dose-response of MPD (0.6, 2.5, and 10.0 mg/kg) on locomotor behavior and sensory evoked potentials recorded from the VTA, NAc, and PFC in freely behaving rats previously implanted with permanent electrodes. For locomotor behavior, adult male Wistar-Kyoto (WKY; n = 39) rats were given saline on experimental day 1 and either saline or an acute injection of MPD (0.6, 2.5, or 10.0 mg/kg, i.p.) on experimental day 2. Locomotor activity was recorded for 2-h post injection on both days using an automated, computerized activity monitoring system. Electrophysiological recordings were also performed in the adult male WKY rats (n = 10). Five to seven days after the rats had recovered from the implantation of electrodes, each rat was placed in a sound-insulated, electrophysiological test chamber where its sensory evoked field potentials were recorded before and after saline and 0.6, 2.5, and 10.0 mg/kg MPD injection. Time interval between injections was 90 min.

**Results:**

Results showed an increase in locomotion with dose-response characteristics, while a dose-response decrease in amplitude of the components of sensory evoked field responses of the VTA, NAc, and PFC neurons. For example, the P3 component of the sensory evoked field response of the VTA decreased by 19.8% ± 7.4% from baseline after treatment of 0.6 mg/kg MPD, 37.8% ± 5.9% after 2.5 mg/kg MPD, and 56.5% ± 3.9% after 10 mg/kg MPD. Greater attenuation from baseline was observed in the NAc and PFC. Differences in the intensity of MPD-induced attenuation were also found among these brain areas.

**Conclusion:**

These results suggest that an acute treatment of MPD produces electrophysiologically detectable alterations at the neuronal level, as well as observable, behavioral responses. The present study is the first to investigate the acute dose-response effects of MPD on behavior in terms of locomotor activity and in the brain involving the sensory inputs of VTA, NAc, and PFC neurons in intact, non-anesthetized, freely behaving rats previously implanted with permanent electrodes.

## Background

Methylphenidate (MPD), also known as Ritalin, is a psychomotor stimulant of the central nervous system (CNS) that has a similar chemical structure to amphetamine and methamphetamine [1-3]. It has been reported that an estimated 20 million monthly prescriptions for analeptic medications were written for the treatment of attention deficit/hyperactivity disorder (ADHD) [4], of which MPD was the most frequently prescribed medication [5,6]. The drug has been shown to block the dopamine transporter (DAT) and thereby elevates extracellular dopamine (DA) levels in the ventral tegmental area (VTA), nucleus accumbens (NAc), and prefrontal cortex (PFC), which are brain areas of the mesocorticolimbic DA system involved in the locomotor and reinforcing effects of psychostimulants and other drugs of abuse [7-13]. A neuroimaging study indicated that MPD shares similar in vivo potency as cocaine in blocking the DAT in human brain [14]. Despite the escalating consumption of MPD [15,16] and the fact that MPD has many of the same neuropharmacological effects as amphetamine and cocaine [14,17], the adverse effects of MPD treatment in children and adults remain controversial. Some studies have correlated ADHD with subsequent substance abuse [18-20], while other studies have reported that pharmacotherapy of ADHD reduces the risk for substance abuse [21,22].

Although MPD potently attenuates the hyperactivity, impulsivity, and inattention in 60–90% of ADHD cases, the optimal dosages of the drug have not yet been established [23,24]. Currently, there is not any empirical data to consistently show linear improvements with dose [25,26] and the adverse effects of high dose levels of MPD [27-29].

To assess differential effects of MPD dosage, it is important to evaluate the effects on the brain itself and on the whole animal, that is, behavior [29]. However, few studies have investigated the neurophysiological properties of psychostimulants, such as MPD, in intact humans or animals [30], especially its behavioral effects and alteration of sensory evoked neuronal activity in brain regions that are involved in the mesocorticolimbic DA system. Most neurophysiological studies that investigated psychostimulants have been conducted in vivo in the presence of anesthesia [4,31,32], which is known to interfere with CNS activity [33], or obtained in vitro on brain slices [34-38]. A valuable method to studying the mechanistic action of psychostimulants, such as MPD, on neuronal population is to record neuronal activity before and after administration of the psychostimulant in an intact, non-anesthetized, freely behaving subject through sensory-evoked field responses following sensory stimulation. During such recording, on-going physiological events occurring before and after drug administration in specific brain sites can be examined without any anesthetic interference. Thus, simultaneous recordings of neuronal activity from brain regions identified as sites for MPD action are very important and warrant further investigations. It provides the opportunity to study information processing in millisecond temporal resolution time locked to the occurrence of the stimulus [39]. The objective of the present study was to determine the acute dose-response effects of MPD on sensory evoked potentials recorded from the VTA, NAc, and PFC following an acoustic stimulus in intact, non-anesthetized freely behaving rats previously implanted with permanent electrodes and correlating these neuronal effects to the rat's locomotor behavior.

## Results

### Behavior – locomotor activity testing

**Figure 1:**
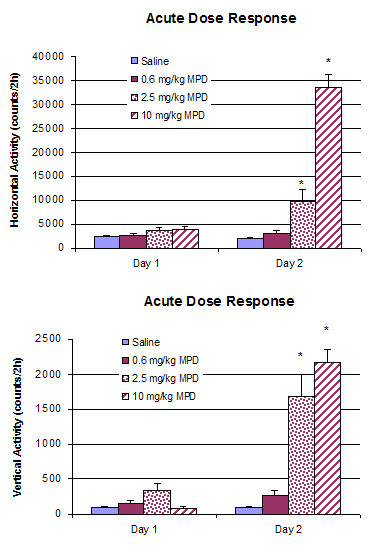
summarizes the dose response of adult male WKY rats to an acute administration of 0.6 (n = 8), 2.5 (n = 8), and 10.0 (n = 8) mg/kg MPD as compared to the control rats that received saline (n = 8). Values are presented as the mean + S.E.M., where at least *p < 0.05.

Figure 1 summarizes the dose response of adult male WKY rats to an acute administration of 0.6, 2.5, and 10.0 mg/kg MPD as compared to the control rats that received saline. Values are presented as the mean + S.E.M., where at least *p < 0.05. There was no significant difference in the horizontal and vertical activities of the saline treated rats on experimental day 1 and day 2. Similarly, there was also no significant difference in these motor indices of rats that received saline on experimental day 1 and 0.6 mg/kg MPD on experimental day 2. However, a single injection of 2.5 mg/kg MPD significantly increased the horizontal [F_1,15 _= 6.459, p = 0.024] and vertical [F_1,15 _= 6.097, p = 0.027] activities on experimental day 2 when compared to saline on experimental day 1. The 10 mg/kg MPD dose elicited a significantly further augmentation (p < 0.01) as compared to the 2.5 mg/kg dose in both of these locomotor indices (horizontal activity [F_1,15 _= 117.77, p = 0.00]; vertical activity [F_1,15 _= 126, p = 0.00]) on experimental day 2 when compared to day 1, on which the rats received saline.

### Sensory evoked potential recording

**Figure 2:**
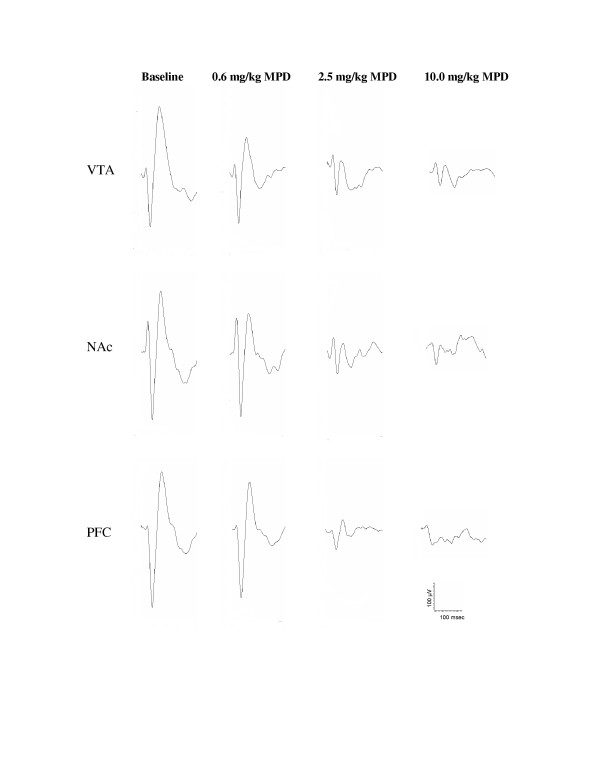
shows a representative of the average (n = 50) sensory evoked field potential responses recorded in the VTA, NAc, and PFC upon acoustic stimulation following the administration of saline (baseline) and acute MPD (0.6, 2.5, and 10.0 mg/kg, i.p.). Time interval between injections was 90 min. All recordings were obtained 20 min post-injection of saline and MPD.

Fifty acoustic evoked responses were averaged. Four such averages were performed before and after each injection. Figure 2 shows a representative of the averaged (n = 50) sensory (acoustic) evoked field potential recorded simultaneously in the VTA, NAc, and PFC following the administration of saline (baseline) and a single injection of 0.6, 2.5, and 10 mg/kg, i.p., MPD. All three MPD doses attenuated the amplitude of the field potential components (P2, N2, and P3) as compared to those of baseline in the VTA, NAc, and PFC neurons. All recordings were obtained 20 min post-injection of saline and MPD. It became evident that the 0.6 mg/kg MPD that failed to alter locomotion attenuated the acoustic average of evoked responses. Moreover, the higher MPD doses that had increased locomotor behavior further attenuated the averaged sensory evoked responses in the VTA, NAc, and PFC (Fig. 2).

**Figure 3:**
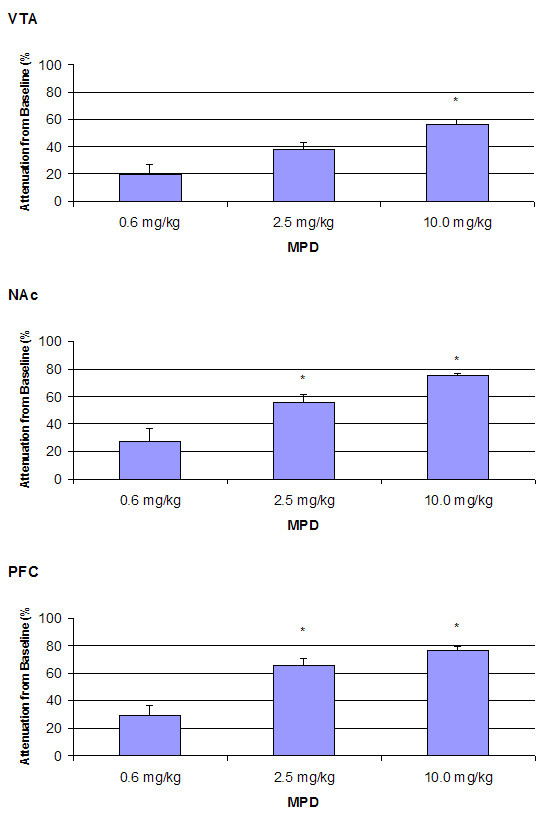
summarizes the percent decrease in amplitude of the sensory (acoustic) evoked responses for component P3 as compared to control recording in the VTA, NAc, and PFC. The control amplitude after saline injection was arbitrarily set as zero. The decrease in amplitude of 0.6, 2.5, and 10.0 mg/kg MPD doses was calculated as the average of four post-injection time points (10, 20, 30, and 40 min) and is presented as the mean + S.E.M, where *p < 0.05 as compared to 0.6 mg/kg MPD.

Figure 3 summarizes the percent decrease in amplitude of the sensory (acoustic) evoked responses for P3 component as compared to control recording in the VTA, NAc, and PFC. The control amplitude after saline injection was arbitrarily set as zero. The decrease in amplitude following 0.6, 2.5, and 10.0 mg/kg MPD administration was calculated as the average of four post-injection time points (10, 20, 30, and 40 min) and is presented as the mean + S.E.M. In the VTA (Fig. 3), a single injection of 0.6 mg/kg, 2.5 mg/kg, and 10.0 mg/kg MPD attenuated the averaged sensory evoked responses in dose response characteristics, i.e., further attenuation was observed with increased dose of MPD. Thus, the higher MPD doses (2.5 mg/kg and 10.0 mg/kg) decreased the amplitude of sensory evoked responses for P3 component more than the 0.6 mg/kg dose (F_2,11 _= 9.784, p = 0.006). Similarly, a single injection of 0.6, 2.5, and 10.0 mg/kg MPD, i.p., also attenuated the averaged sensory evoked responses of P3 component recorded from the NAc and PFC in dose response characteristics (Fig. 3). Similar attenuation following 0.6, 2.5, and 10.0 mg/kg MPD were obtained for P2 and N2 components (Table 1).

**Table 1:** The percent decrease in amplitude from baseline of P2 and N2 components in the various brain areas of adult male WKY rats following a single injection of 0.6, 2.5, and 10.0 mg/kg MPD (i.p.).

**Component**	**Brain Area**	**0.6 mg/kg MPD**	**2.5 mg/kg MPD**	**10.0 mg/kg MPD**
**P2**	VTA	15.3 ± 5.5%	38.8 ± 6.2%	41.3 ± 10.6%
	NAc	12.5 ± 9.2%	20.5 ± 2.7%	51 ± 5.9%
	PFC	-11 ± 2.2%	34.3 ± 4.7%	43 ± 8.7%

**N2**	VTA	21.8 ± 5.9	34.8 ± 6.7%	55 ± 6 %
	NAc	23.5 ± 5.9%	53.5 ± 3.2%	60 ± 2.4%
	PFC	29.5 ± 7.4	65 ± 6	76.5 ± 2.7

**Figure 4:**
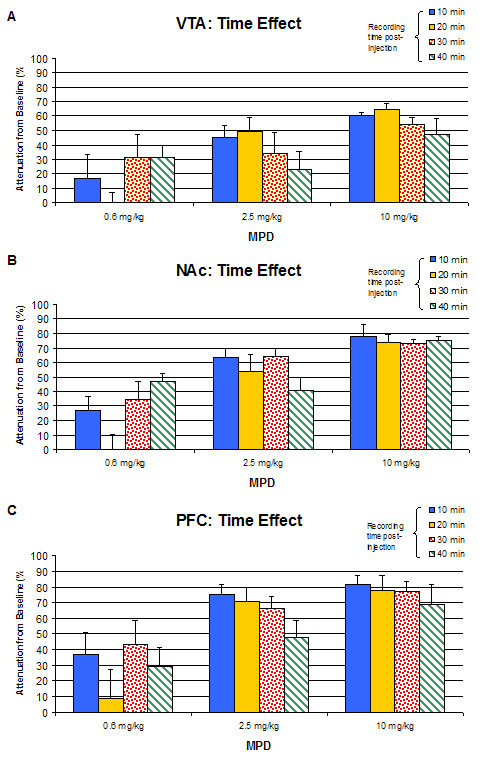
summarizes the effect of 0.6, 2.5, and 10.0 mg/kg MPD in the component P3 of VTA, NAc, and PFC at 10, 20, 30, and 40 min post-injection. Values are presented as the mean + S.E.M.

Figure 4 summarizes the effect of 0.6, 2.5, and 10.0 mg/kg MPD for the P3 component of the sensory evoked responses recorded from the VTA, NAc, and PFC at 10, 20, 30, and 40 min post-injection. Values are presented as the mean + S.E.M. In general, there was not any significant time effect found in all three brain regions following the administration of 0.6, 2.5, and 10.0 mg/kg MPD. All four post-injection time points exhibited similar attenuation in amplitude from baseline following the administration of each MPD dose in the VTA, NAc, and PFC. There was also no time effect (e.g., recovery) found in P2 and N2 components of the averaged sensory evoked responses (Table 2).

**Table 2:** The effect of a single administration of 0.6, 2.5, and 10.0 mg/kg MPD on the attenuation in amplitude from baseline of P2 and N2 components during 10, 20, 30, and 40 min post-injection.

		VTA			NAc			PFC		
	Time post-injection	0.6 mg/kg	2.5 mg/kg	10.0 mg/kg	0.6 mg/kg	2.5 mg/kg	10.0 mg/kg	0.6 mg/kg	2.5 mg/kg	10.0 mg/kg

P2	10 min	11.0	57.0	58.0	21.0	17.0	56.0	-9.0	21.0	63.0
	20 min	3.0	36.0	57.0	-12.0	28.0	61.0	-11.0	38.0	52.0
	30 min	29.0	32.0	37.0	10.0	16.0	53.0	-7.0	43.0	28.0
	40 min	18.0	30.0	13.0	31.0	21.0	34.0	-17.0	35	29

N2	10 min	16.0	51.0	68	34.0	62.0	64.0	37.0	75.0	82.0
	20 min	4.0	40.0	61.0	11.0	48.0	61.0	9.0	71.0	78.0
	30 min	37.0	26.0	50.0	16.0	55.0	62.0	43.0	66.0	77.0
	40 min	30.0	22.0	41.0	33.0	49.0	53.0	29.0	48.0	69.0

**Figure 5:**
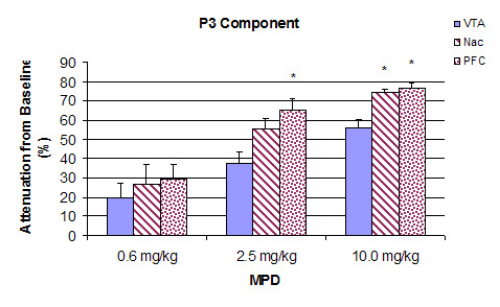
summarizes and compares the acute dose response characteristics of component P3 of the VTA, NAc, and PFC following the administration of 0.6, 2.5, and 10.0 mg/kg MPD. Values are presented as the mean + S.E.M., where * p < 0.05 as compared among VTA, NAc, and PFC.

Figure 5 summarizes and compares the acute dose response characteristics of component P3 of the VTA, NAc, and PFC following the administration of 0.6, 2.5, and 10.0 mg/kg MPD. Values are presented as the mean + S.E.M., where * p < 0.05 as compared among VTA, NAc, and PFC. The 0.6 mg/kg dose attenuated the sensory evoked responses in all three sites, and the intensity of the attenuation was similar in all of them. However, for example, following the 2.5 mg/kg dose, the P3 component from the PFC exhibited the most attenuated effect (65%; F_2,11 _= 5.848, p < 0.05) compared to the VTA (37.8%) and NAc (55.5%). As the MPD dosage increased to 10.0 mg/kg, the P3 component further decreased by 56.5%, 75.0%, and 76.5% from the VTA, NAc, and PFC recordings, respectively, at a significant level (F_2,11 _= 15.73, p < 0.05). Similar observations were obtained for the P2 and N2 components of the average sensory evoked responses recorded from the VTA, NAc, and PFC neurons (Table 1).

## Discussion

There are different approaches to assess the effects of psychostimulants on animals. This study reports two approaches – behavior and neurophysiology. Sunohara et al. [29] proclaimed that it is important to use both behavioral and neurophysiological approaches. It is known that psychostimulants modulate the expression of the subjects' behavior via alteration of their sensory input. In the present study, we report behavioral as well as electrophysiological observations following sensory stimulation before and after several doses of the psychostimulant MPD. Most of the electrophysiological experiments investigating the property of MPD have been conducted in the presence of anesthesia [4,31,32], which is known to modulate CNS activity [33], or used brain slices [34-38] to record the effect of the drug on spontaneous activity and the role of different neurotransmitters in MPD action. None of the studies investigate, in freely behaving animals previously implanted with permanent electrodes and without the interference of anesthesia, the dose-response characteristics on sensory input recorded simultaneously from sites where psychostimulants are known to initiate and/or express their effects. Thus, the objectives of the present study were to investigate the acute dose-response effects of MPD on animal behavior, as well as on sensory evoked potentials from the brain areas considered to be the sites of psychostimulant action, such as the VTA, NAc, and PFC, in intact, non-anesthetized, freely behaving rats previously implanted with permanent electrodes. Thoughts were given to correlate the effects of the drug on animal behavior to its neurophysiological responses in specific brain areas. The present study reports four main findings: (1) single 0.6 mg/kg MPD failed to alter locomotor activity, while 2.5 and 10.0 mg/kg MPD injection elicited increase in locomotor activity in a dose response manner; (2) the amplitude of the average sensory evoked response components (P2, N2, and P3) obtained from the VTA, NAc, and PFC exhibited the opposite effects from the behavior, that is, MPD elicited dose-response attenuation to all three MPD doses in a dose-response characteristics; (3) differences in the MPD-induced attenuation of P2, N2, and P3 amplitudes were found among VTA, NAc, and PFC; and (4) there was not any significant time effect in the attenuation of P2, N2, and P3 amplitudes following administration of 0.6, 2.5, and 10.0 mg/kg MPD during the 40 min post drug administration.

In the behavioral dose-response experiment, the lowest MPD dose used (0.6 mg/kg) failed to produce any significant effect on the locomotor responses of the rats compared to saline (Fig. 1). However, a single administration of 2.5 mg/kg MPD elicited significant augmentation in the horizontal and vertical activities. The 10.0 mg/kg MPD induced further increase of these locomotor behaviors compared to 2.5 mg/kg MPD. The present behavioral observation confirms and extends previous reports of increased locomotor activity in response to a single MPD administration [40-44]. Increased motor activity is the most commonly observed effect of psychostimulants in animal models. It is an integrated response that represents the final expression of different neuronal processes.

The same MPD doses elicited the opposite effects on the sensory inputs recorded from the VTA, NAc, and PFC areas. The amplitude of the sensory responses consists of P1, N1, P2, N2, and P3 components. The cognitive processes and their underlying mechanisms associated with each of these components have not been definitively identified. However, sensory evoked potential recording is an approach frequently used for analyzing information processing in the human brain [45] because these components reflect cognitive processes at specific, neuroanatomical locations [29,46]. For examples, P2 reflects feature detection and is observed at the central and frontal areas of the brain [47]; N2 reflects target identification when it is observed at anterior locations of the brain [48]; and P3 reflects further processing and evaluation of the relevant stimulus [49] and updating working memory and post decisional processes [45]. It is largest over parietal and central regions of the brain [45].

In general, all MPD doses attenuated the three amplitudes (P2, N2, and P3) of the AAER in dose-response characteristics, i.e., increasing the MPD doses further attenuated the amplitude, except for the P2 component recorded from the PFC following 0.6 mg/kg administration. This observation supports the findings by Arnsten and Dudley [50] that low MPD dose improved PFC cognitive function. In contrast to the non-significant change in locomotor activity observed after 0.6 mg/kg MPD treatment compared to that of saline, acute administration of 0.6 mg/kg MPD (i.p.) attenuated the amplitude of P2, N2, and P3 components of sensory evoked field responses from the VTA, NAc, and PFC when compared to that of saline baseline (Fig. 2). It appears that this MPD dosage attenuated the average acoustic evoked responses by 19.8%, 27%, and 29.5% in the VTA, NAc, and PFC, respectively, while the same MPD dose (0.6 mg/kg) was not efficacious enough to elicit a behavioral (locomotor) response. This attenuation of sensory input persisted and became more pronounced as the MPD dosage increased to 2.5 and 10.0 mg/kg (Fig. 3), while locomotor activity following these MPD doses significantly increased with dose-response characteristics (Fig. 1).

Methylphenidate elicits its effects on the CNS via the mesocorticolimbic system. Therefore, sensory evoked potential responses were recorded in these brain regions. The mesocorticolimbic pathway consists of DA neurons in the VTA that project to the NAc and PFC [51]. Methylphenidate binds to DAT at the presynaptic region of these brain areas and blocks their DA re-uptake, resulting in accumulation of extracellular DA [52]. The extracellular DA act at both presynaptic and postsynaptic receptors. The activation of presynaptic DA autoreceptors expressed by the somatodendritic regions of DA neurons exerts a strong inhibitory effect on the neuronal activity and transmitter release of these neurons [53]. Thus, increased extracellular DA level produces inhibition of the firing of DA neurons [54-57] and may result in further attenuation from baseline of P2, N2, and P3 components of VTA, NAc, and PFC neurons as the MPD dosage increased in the present study. The presence of a negative feedback mechanism involving increased extracellular DA is also confirmed by a study showing that when the DA-mediated feedback inhibition was blocked by raclopride, a DA antagonist, the psychostimulant d-amphetamine, instead of producing no effect, effectively excited the DA cells [58]. Increased extracellular DA concentration also activates postsynaptic DA receptors and thereby enhances the motor-activating effects [53], as observed in the behavioral dose-response of MPD (Fig. 1).

An acute administration of d-amphetamine has also been shown to excite some DA neurons recorded in the VTA [59]. It may be that some DA neurons in the VTA, NAc, and PFC of non-anesthetized rats exhibit two types of DA receptors, that is, receptors that the DA causes excitation and other receptors that the DA causes inhibitory effects in response to psychostimulants but that the average of these two results in an inhibitory effect. This may lead to a plausible explanation for the differences in the MPD-induced attenuation of N2 and P3 amplitudes among the VTA, NAc, and PFC (Fig. 5). That is, differential populations of DA neurons that respond to DA by excitation or inhibition are among the VTA, NAc, and PFC which affect the net outcome. It is possible that this attenuation of sensory input inhibits the inhibitory component (i.e., disinhibition) of the motor system and the motive circuit nuclei, which results in locomotor activation and improves cognition.

The sensory evoked responses were obtained from 10 to 40 min post injection of MPD. This window for recording was based on our previous behavioral experiment [44], showing that the peak effect on locomotion was around 10 min and the duration was about 40 min. To our surprise, the MPD effects on the sensory input in all of the brain sites recorded remained the same during the entire recording time (Fig. 4), suggesting that the sensitivity to express effect of a drug between behavior and electrical activity is different. Moreover, if there is effect on neuronal activity, it is not necessary that the animal's behavior expresses the drug effects.

## Conclusion

Collectively, these results suggest that an acute treatment of MPD produces electrophysiologically detectable alterations at the neuronal level, as well as observable, behavioral responses. It is believed that through systematic analysis of waveforms investigators can follow attentional processing from the early stages of initial stimulus detection to subsequent mental representation and response execution [29]. To our knowledge, the present study is the first to investigate the acute dose-response effects of MPD on behavior in terms of locomotor activity and in the brain involving the sensory inputs of VTA, NAc, and PFC neurons in intact, non-anesthetized, freely behaving rats previously implanted with permanent electrodes. Given the fact that repeated exposure to psychostimulants, such as amphetamine, cocaine, and methamphetamine, results in elicited augmented behaviors known as behavioral sensitization [13,60,61] and a variety of neuroadaptating processes associated with addiction [62-64], it is essential that further studies on MPD through sensory evoked potentials recordings are necessary in order to gain further knowledge on the mechanism of MPD action and how the drug modulates the ADHD patient's sensation to alter his or her behavior, as well as the mechanisms that correlate to locomotor activity and attentional processing, especially in terms of chronic administration of MPD, behavioral sensitization, and drug dependence.

## Methods

### Subjects

Adult male Wistar-Kyoto rats (n = 49; 65–70 postnatal days) were obtained from Harlan (Indianapolis, IN, USA). Upon receipt, these rats weighed 260–270 g. They were housed in groups of two per Plexiglas cage and maintained on a 12-h light/dark cycle (lights on from 05:30 to 17:30 h) with an ambient temperature of 21 ± 2°C and a relative humidity of 37% to 42%. Rats were given food pellets and water ad libitum throughout the study. They acclimated in this room for one week prior to any experimental manipulation. All experiments were carried out in accordance with the National Institutes of Health Guide for the Care and Use of Laboratory Animals and our institution's Animal Welfare Committee Guidelines.

### Drugs

Methylphenidate hydrochloride (MPD) was a gift from Mallinckrodt Inc. (St. Louis, MO, USA). There are not universally recognized dosage guidelines and blood levels to achieve optimal treatment with MPD [65]. A study of 289 patients treated with MPD reported that the range of doses ingested in these patients was from 0.06 – 29.3 mg/kg and that the majority of the patients were treated with 1.0 – 3.0 mg/kg MPD [66]. Generally, intraperitoneal (i.p.) administration leads to peak plasma level of MPD much higher and faster than oral administration. The binding of MPD to dopamine transporter (DAT) increases DA in the synaptic cleft in intraperitoneal administration [67,68]; while oral MPD application elevates mainly norepinephrine (NE), in addition to DA [68], which improves cognitive function through α2 adrenoceptors and dopamine D1 receptor actions [50] without increasing locomotion. Drug effects in rodents often require higher doses on an mg/kg basis than in humans because rodents exhibit a more rapid metabolism [52]. In selecting the equivalent dose for rats as compared to dosage in humans, we must take into account these differences in the pharmacokinetics between humans and rodents, which include route of administration, absorption, volume, metabolism, and excretion [67,69,70].

In our preliminary behavioral experiment, we used 0.1, 0.6, 1.2, 2.5, 5.0, 10.0, 20.0, and 40.0 mg/kg MPD (i.p.). The three initial MPD doses (0.1, 0.6, and 1.2 mg/kg) did not exhibit any effect on locomotor activity, while the 2.5 mg/kg dose increased locomotion in dose-response characteristics [42]. Therefore, in the present study, we selected three MPD doses. The first is 0.6 mg/kg, which does not exhibit any effect in locomotor activity. The second and third doses are 2.5 and 10.0 mg/kg, which fall within the clinical range in treating children and adult with ADHD [50,52,60,61,67,68]. The drug was dissolved in 0.9% saline, and the dosages were calculated as free-base. All injections were administered intraperitoneally (i.p.) between 07:00 h and 12:00 h and equalized to a volume of 0.8 ml with 0.9% saline so that the total volume of each injection in all animals would be the same.

### Behavioral experiments

#### Protocol

Rats (n = 39) were randomly divided into four different treatment groups (saline, 0.6, 2.5, or 10.0 mg/kg MPD). Each rat was individually placed inside an automated activity monitoring cage located in the sound-attenuated, temperature-, and humidity-controlled room to habituate for 24 h prior to the first experimental day (Day 1). The activity monitoring cage now served as the animal's home cage for the next two days. All rats received an injection of 0.9% saline on experimental day 1. On experimental day 2, Groups I (n = 8), II (n = 11), III (n = 12) and IV (n = 8) rats received saline, 0.6, 2.5, and 10.0 mg/kg MPD, respectively. Locomotor activities were recorded for 2 h following saline/drug administration on each of the two experimental days.

#### Apparatus

Each activity monitoring cage (Accuscan, Columbus, OH, USA) consisted of a clear, acrylic open-field box (40.5 × 40.5 × 31.5 cm) fitted with two levels of infrared motion sensors located 6 and 12.5 cm above the floor of the box. This system checked for interruptions of each infrared beam at a frequency of 100 Hz. Interruption of any beam was recorded as an activity score. Simultaneous interruptions of two or more consecutive beams separated by at least 1 sec were recorded as a movement. Cumulative counts were compiled and downloaded every 10 min into the OASIS data collection software that organized and differentiated these counts into various locomotor indices.

Two locomotor indices were evaluated: horizontal activity and vertical activity. Horizontal activity measures the total number of beam interruptions that occurred at the lowest tier (horizontal sensor) during a given sample period. Vertical activity measures the total number of beam interruptions that occurred in the vertical sensor (upper tier) during a given sample period which counted mainly rearing.

#### Data analysis

The saline treated rats served as the control group. In addition, each animal served as its own control. Therefore, in addition to examining and comparing the cumulative drug effects observed between treatment groups, the responses of the animals to the drugs were also evaluated by having each animal within a treatment group served as its own control to eliminate any handling and injection effects. This was determined by subtracting the activity score of the 2-h after saline injection (experimental day 1) from that of the 2-h after drug injection on experimental day 2. Thus, the activity of saline injection served as the baseline and the absolute change in activity from saline represented the effect of the drugs. In a previous study [44], it was found that the duration of MPD on locomotion was 50–90 min; therefore, a 120 min cumulative activity was recorded after each treatment. Observations within a treatment group and between treatment groups were analyzed using Analysis of Variance (ANOVA: treatment days and drug dose) and post-hoc Fischer's LSD method. Statistical significance was set at p < 0.05 for all comparisons.

### Neurophysiological experiments

#### Surgical procedure

After 3–7 days of acclimation upon arrival from the vendor, adult male WKY rats (n = 10) were anesthetized with 50 mg/kg pentobarbital (i.p.) and placed in a stereotaxic apparatus. A 2 cm skin incision was made, and the skull was exposed. A hole of 1 mm in diameter was drilled to insert an electrode into selected brain areas. Brain coordinates derived from Paxinos and Watson [71] atlas were used to implant stainless steel semi-microelectrodes (80 μm in diameter) bilaterally in the prefrontal cortex (PFC: Bregma 2.7 mm, lateral 0.6 mm, depth 3.8 mm), nucleus accumbens (NAc: Bregma 1.7 mm, lateral 1.6 mm, depth 6.8 mm), and ventral tegmental area (VTA: Bregma -4.8 mm, lateral 1.0 mm, depth 8.3 mm). An electrode was also implanted in the nasal skull as a reference. All electrodes were fixed permanently to the skull with dental acrylic cement and attached to terminals in an Amphenol plug.

#### Sensory evoked field potentials

Two days after recovery from the implantation of electrodes, rats were placed inside a cubic Plexiglas box (23 cm^3^) located within a sound-insulated, electrophysiological test chamber for habituation to the testing environment. Each rat received a daily 30-min habituation period in this box for two consecutive days prior to the experimental day.

On experimental day (5 to 7 days after electrode implantation), the rat was put back inside the Plexiglas box to habituate for 30 min prior to experimentation. The electrodes in the rat's head were encased in an Amphenol plug, which was connected to a Grass P511 amplifier by means of low noise leads via a commutator mounted on a counter-balanced arm that allowed the animal to move freely in the Plexiglas box. From the amplifier, the neuronal activities were monitored with multi-beam oscilloscopes and connected to the Micro 1041 (Cambridge Electronic Design, Cambridge, England). The Micro 1041 was connected to a PC computer equipped with the Spike 2 program (Cambridge Electronic Design, Cambridge, England) for digitizing, averaging, and storing the data for off-line evaluation. Acoustic stimulation was in the form of 'clicks' produced by a Grass AC-5 audio-stimulator. The remote speaker (10 cm in diameter) was placed 1.0 cm from the Plexiglas box. A digitimer device (model # 3290) triggered the acoustic stimulator and all other equipments. At every 2.4 second, a stimulus was presented. Four trains, each consisted of 50 acoustic stimuli, were presented at 5-min intervals for control (saline) recordings and resumed every 10-min after each drug injection for an additional four trains of 50 acoustic stimulations. Each stimulating session lasted 2 min (50 stimuli every 2.4 sec = 120 sec = 2 min). Sensory evoked field potentials were simultaneously recorded from the VTA, NAc, and PFC after saline, 0.6, 2.5, and 10 mg/kg MPD injection (i.p.). After saline injection and control recording, each animal was injected with 0.6, 2.5, and 10.0 mg/kg. Time between injections was 90 min. This time interval was based on our previous behavioral experiment [44] that demonstrated that after 50 to 70 min after the administration of 2.5 and 10.0 mg/kg MPD the locomotor activity returned to baseline. Therefore, in the present study, we used 90 min between injections to reduce the effects of the previous dose.

#### Data analysis

Fifty sensory evoked responses were averaged off-line using the Spike 2 program (Cambridge Electronic Design, Cambridge, England). For every acoustic stimulus, four averages were calculated after every saline/MPD injection. Each averaged sensory evoked response was evaluated in terms of amplitude of the characteristic components from peak to peak. The acoustic sensory evoked response on a rat exhibited five main components: P1, N1, P2, N2, and P3. The 'P' indicates positive amplitude; the 'N' indicates negative amplitude. The integers indicate the first, second, or third component, i.e., P1 refers to the first positive component of the evoked field response amplitude. The P1 and N1 components were not consistent within and between animals and therefore were not analyzed. The P2, N2, and P3 components were the most consistent components within and between animals in the three selected brains areas of this study and therefore were evaluated. Changes induced by the three doses of MPD were evaluated by comparing the component amplitudes averaged following drug injection recording to that of the control (saline) recording. A mean amplitude change ± 2 standard error (either increase or decrease) in comparison to the control recording was regarded as a significant change [72,73]. Each animal served as its own control. Comparison between groups was performed with ANOVA with 95% confidence level.

#### Histological verification of electrode placement

At the end of the experiment, rats were overdosed with 200 mg/kg sodium pentobarbital. A lesion was placed at the tip of each electrode by passing a 50 μA direct current for 30 seconds. The brain was then transcardially perfused with a solution of 10% formalin containing 3% potassium ferrocyanide. Brain sections were cut serially at a thickness of 80–100 μ using a vibrotome (OTS-3000-03; FHC, Brunswick, ME, USA) and stained with Cresyl violet. The position of the electrode tips was identified by the location of the lesion and Prussian blue spot.

## Authors' contributions

The experiment was conceived, developed, and reported collaboratively by all authors. PBY was primarily responsible for data collection and analysis.
